# Diabetes care: reasons for missing HbA_1c _measurements in general practice

**DOI:** 10.1186/1756-0500-4-234

**Published:** 2011-07-14

**Authors:** Debby G Keuken, Henk J Brouwer, Parad S Keijser, Raynold C Bruessing

**Affiliations:** 1 Department of General Practice, Division of Clinical Methods & Public Health, Academic Medical Center-University of Amsterdam, Amsterdam, Netherlands; 2 Stichting Gezondheidscentra Amsterdam Zuidoost, Amsterdam, Netherlands

## Abstract

**Background:**

Glycated haemoglobin (HbA_1c_) is often used as one of the indicators to measure the quality of diabetes care. Complete registration is difficult to obtain. This study investigated the reasons for missing HbA_1c _measurements.

**Findings:**

HbA_1c _measurements for 1485 patients with diabetes mellitus type 2 who were attended by 19 general practitioners at 4 primary care health centres in south-east Amsterdam were studied. HbA_1c _measurements were missing for 356 (23.9%) of the patients. The main reason stated in 50% of the cases was that the patient was under specialized care.

**Conclusions:**

The general practitioners provided multiple reasons for the missing HbA_1c _measurements. This study provides insight into why HbA_1c _measurements were not present in the patients' electronic medical record.

## Background

Dutch guidelines for GP practice contain systematic prescriptions for diabetes type 2 care that include quarterly monitoring of the lifestyle of diabetes patients by practice nurses and annual evaluations of optimal glycaemic control by GPs. The guidelines emphasize annual evaluation of micro-vascular complications, as well as cardiovascular risk factors and vascular damage [1]. The identification of barriers impeding adherence to guidelines has proven a necessary condition for the further improvement of care strategies [2-4]. The measurement of glycated haemoglobin (HbA_1c_) is an essential part of the annual evaluation of glycaemic control for patients with diabetes type 2 [1,5].

In several countries (e.g. the UK and the Netherlands), GP practices are required to obtain a certain number of measurements and outcomes as guideline-based diabetes care indicators for pay-for-performance purposes [6-8]. Although indicators in the Netherlands are standardized nationally [9,10], performance indicators are organized in local settings based on contracts with health insurance companies. The effects of quality and outcome frameworks have been evaluated and described [11,12], however, these studies do not provide reasons for missing indicator measurements.

To improve understanding regarding non-adherence to the guideline-based care, we formulated the following research questions:

1) How complete are HbA_1c _measurement data?

2) Which factors can explain missing data?

## Methods

### Setting

We used a subset of data from the GP registration network of the department of General Practice of the Academic Medical Center-University of Amsterdam (AMC). The network uses routinely recorded data in electronic medical records for research purposes. Diagnoses are coded according to the International Classification of Primary Care (ICPC). Four health centres in south-east Amsterdam participate in this network. At these health centres, 22 GPs and 8 practice nurses provide primary care to 35,700 patients. The four centres belong to the same umbrella organization (GAZO), which signs the pay-for-performance contract with a health insurance company for standard, structured multidisciplinary diabetes care under GP coordination. Since 2007, the registration of diabetes care indicators has been implemented within this network as part of a disease management programme that includes feedback sessions. This registration was implemented in order to enhance efforts to improve the quality of diabetes care and to provide an infrastructure for diabetes-related research. All of the health centres use the same computerized patient electronic medical record system (MIRA). Patients with diagnosed diabetes type 2 are coded ICPC T90.2 in their electronic medical records. Only patients whose were under structural care by the GPs were included in our study.

Following regular care, patients visit their health centres every three months for lifestyle consultations, medication evaluations and evaluations of their well-being, and annually for comorbidity screening and therapy planning. A minimum of two weeks before the annual visits, HbA_1c _blood levels are measured in a blood laboratory. Results of these measurements are forwarded electronically to the patients' electronic medical records.

### Identification of reasons for non-registration

The number of practice-based measurements of the diabetes-care indicator HbA_1c _was calculated over the year 2007. This 12-month period is in line with the regional pay-for-performance contract. Nineteen of twenty-two practices at the four health centres agreed to participate in this study. The number of measurements was presented to these practices during feedback sessions. After the sessions, each GP was given a form with the numbers referring to patients for whom HbA_1c _measurements were missing. The GPs were asked to state the main reason for each missing HbA_1c _measurement.

The stated reasons were collected and grouped into categories by one of the authors (HJB). This grouping was presented to the other authors. Disagreements were resolved through discussion.

## Results

In 2007, 1485 patients diagnosed with diabetes type 2 visited the participating GPs (n = 19). For 356 (23.9%) of these patients, HbA_1c _measurements were absent over a 12-month calendar year period. Table 1 lists the reasons for missing HbA_1c _measurements, grouped into eight categories. In 50% of the cases, the stated reason is that the patient is under treatment by a specialist (reason 1). Reasons 2 and 3 (both 7%) are inherent to the chosen timeframe of one calendar year. Reason 4 (3%) is caused by the administrative process. Reasons 5 and 6 (8% and 3%, respectively) are patient related. The missing HbA_1c _measurements attributed to reason 7 (7%) are due to misclassification of the diagnoses of patients with pre-diabetes conditions (hyperglycaemia, gestational diabetes). Reason 8 (6%) refers to practical problems in performing regular patient monitoring. Reason 9 (6%) comprised 'other' reasons (e.g. dementia or recent registration at the practice). In 3% of the cases, the GP provided no reason. The practices of the non-participating GPs (n = 3) did not have higher rates of missing HbA_1c _measurements (mean: 21%, range: 15-32%) than did the participating GP practices (mean: 25%, range: 13-50%).

**Table 1:** Reasons for missing HbA_1c _measurements

	**Reasons for missing HbA**_**1c **_**measurements (n = 355)**	n (%)
1	Patient under specialized care	178 (50%)

2	Patient received first diagnosis of diabetes type 2 within the 12-month period	25 (7%)

3	Patient's HbA_1c _was measured outside the 12-month period	25 (7%)

4	Patient moved or died without this being registered	11 (3%)

5	Patient did not report for HbA_1c _measurement at the blood lab	28 (8%)

6	Patient abroad at time of HbA_1c _measurement appointment	11 (3%)

7	Patient falsely diagnosed or coded diabetes type 2	25 (7%)

8	Patient not invited within 12-month period for HbA_1c _measurement	21 (6%)

9	Other reason	21 (6%)

10	No reason stated by GP	11 (3%)

	**Total**	**356 **(100%)

## Discussion

The GPs provided multiple reasons for missing HbA_1c _measurements. The majority of the missing values are due to patients being in specialized care. A true reflection of the well-being of a population calls for a set of measurements that is as complete as possible, regardless of whether the goal is to obtain data for performance indicators or to conduct scientific research.

Our study provides insight into why HbA_1c _measurements were not present in the patients' electronic medical records when calculating the percentage of available measurements over a 12-month period. Several factors underlie the stated reasons for the missing measurements. Evidence emerged to suggest discrepancies in the diagnostic coding of patients with hyperglycaemia or gestational diabetes (reason 7). In some cases, these conditions were registered as diabetes type 2, which prompted the monitoring system to generate reminders to provide these patients with structural screening for diabetes type 2. The practical implementation of a guideline should be well supported by a logistical system for regular patient appointments. In some cases, this system did not work properly (reason 8). Lack of patient compliance with structural care was absent in some cases (reason 5 and 6). In other cases, there were no clear agreements concerning changes in a patient's data (e.g. due to death or relocation) and how these changes should be recorded (reason 4). If the focus of data collection is a 12-month period, as prescribed by the guideline, newly diagnosed patients and patients whose HbA_1c _measurements were made just outside the 12-month period fall outside the scope of this registration. Also, patients who are under specialized care but not defined as such also fall outside this scope. Due to the use of different electronic medical systems, the health records of patients who are under specialized diabetes care are not uploaded into the GP system. There is currently no way for GP practices or researchers to extract the data that are necessary for monitoring these patients. Because reasons 1 to 4 are currently inevitable, efforts to improve the registration of HbA1c measurements will not be able to increase the percentage to more than 84% of all diabetic type 2 patients registered at GP practices.

Since the implementation of the Quality and Outcome Framework in the UK, several studies have been published describing evaluations [11,12] that were carried out in order to optimize the effects of the framework. Improvements in care, however, depend upon the ability to identify the completeness of registration sets, as well as the underlying causes for missing registrations [2]. Recent description of diabetes process analysis for one UK practice provided neither a description nor an explanation for the missing data [13]. Overcoming barriers to registration may improve both adherence to the guideline and HbA_1c _measurements.

This study had several limitations. The results are based on data provided by 19 of 22 GPs at 4 health centres, with 'No time available' noted as a reason for non-participation. Nonetheless, the HbA_1c _registrations of the non-participating practices did not differ from those of the participating practices. In addition, our results are built on a single cross-section of data concerning a patient population living in a limited geographical area; the south-east Amsterdam. The population in this area comprises patients of various ethnic backgrounds and a high percentage of low-income groups. In 2009, 72% of this population was made up of first or second-generation migrants (i.e. either they or at least one of their parents were not born in the Netherlands) [14], whilst countrywide this was 20% [15]. In 2006, 32% of the households were in the lowest income group [14]. These characteristics might explain why the percentage of HbA_1c _measurements not comparable to corresponding percentages observed in the UK (e.g. 94% described by Tahrani [13]) after the implementation of the Quality and Outcome framework. However, the percentage is comparable to that observed in other parts of the Netherlands (e.g. 84% described by Voorham for the Dutch GIANTT project [16]). Finally, GP care in the Netherlands is arranged differently than it is in other countries, thus possibly influencing the relative availability of HbA_1c _measurements.

## Conclusions

We believe that GPs in several countries will recognize the various reasons we have enumerated for missing HbA1c measurements and the existence of barriers to registration. This information will be useful to GPs, not only in their efforts to implement quality procedures related to diabetes, but also as a way of showing insurance companies why it is not always possible to reach the required minimum registration threshold. The limitations we have identified can be used to improve guideline adherence and the quality of indicator registration.

## Competing interests

The authors declare that they have no competing interests.

## Authors' contributions

DGK, HJB, PSK and RCB designed the study. HJB collected and interpreted the data. HJB performed the analysis, in discussion with DGK, PSK and RCB. DGK drafted the manuscript. All authors were involved in the revision and final approval of the manuscript.
